# Residual Disease after Operative Hysteroscopy in Patients with Endometrioid Endometrial Cancer Associated with Polyps

**DOI:** 10.1055/s-0040-1719145

**Published:** 2021-01-29

**Authors:** Marcelo Simonsen, Henrique Mantoan, Carlos Chaves Faloppa, Lillian Yuri Kumagai, Levon Badiglian Filho, Andrea Guerreiro Machado, Najla Mohamed Tayfour, Glauco Baiocchi

**Affiliations:** 1 Department of Gynecologic Oncology, Instituto de Assistência Médica ao Servidor Público Estadual de São Paulo, São Paulo, SP, Brazil; 2 Department of Gynecologic Oncology, Hospital AC Camargo, São Paulo, SP, Brazil

**Keywords:** endometrial neoplasm, hysteroscopy, endometrial polyp, câncer de endométrio, histeroscopia, pólipo endometrial

## Abstract

**Objective** To evaluate the presence of residual disease in the uterine specimen after hysteroscopic polypectomy or polyp biopsy in patients with endometrioid endometrial cancer (EC).

**Methods** We analyzed a series of 104 patients (92 cases from the Hospital AC Camargo and 12 from the Hospital do Servidor Público Estadual de São Paulo) with polyps that were diagnosed by hysteroscopy, showing endometrioid EC associated with the polyp or in the final pathological specimen. Patients underwent a surgical approach for endometrial cancer from January 2002 to January 2017. Their clinical and pathological data were retrospectively retrieved from the medical records.

**Results** In 78 cases (75%), the polyp had EC, and in 40 (38.5%), it was restricted to the polyp, without endometrial involvement. The pathologic stage was IA in 96 cases (92.3%) and 90 (86.5%) had histologic grade 1 or 2. In 18 cases (17.3%), there was no residual disease in the final uterine specimen, but only in 9 of them the hysteroscopy suggested that the tumor was restricted to the polyp. In 5 cases (4.8%) from the group without disease outside of the polyp during hysteroscopy, myometrial invasion was noted in the final uterine specimen. This finding suggests the possibility of disease extrapolation through the base of the polyp.

**Conclusion** Patients with endometrioid EC associated with polyps may have the tumor completely removed during hysteroscopy, but the variables shown in the present study could not safely predict which patient would have no residual disease.

## Resumo

**Objetivo** Avaliar a presença de doença residual no exame anatomopatológico definitivo de pacientes com câncer de endométrio endometrioide após polipectomia ou biópsia de pólipo histeroscópica.

**Métodos** Analisamos 104 pacientes (92 casos do Hospital AC Camargo e 12 casos do Hospital do Servidor Público Estadual de São Paulo) com pólipos diagnosticados durante histeroscopia e cuja biópsia histeroscópica ou exame patológico final do útero acusaram câncer de endométrio endometrioide. As pacientes foram submetidas a cirurgia para câncer de endométrio de janeiro de 2002 a janeiro de 2017. Os dados clínicos e anatomopatológicos de cada paciente foram retirados dos prontuários médicos

**Resultados** Em 78 casos (75%), o pólipo continha a neoplasia, e em 40 (38.5%), ela estava restrita ao tecido do pólipo, sem envolvimento endometrial adjacente. O estadio final foi IA em 96 casos (92.3%) e em 90 (86.5%) tratava-se de grau 1 ou 2. Em 18 casos (17.3%), não havia doença residual no espécime uterino, mas em apenas 9 deles a histeroscopia sugeriu doença restrita ao pólipo. Em 5 casos (4.8%), não havia doença aparente extrapólipo na histeroscopia, mas havia invasão miometrial, sugerindo extravasamento do tumor pela base do pólipo.

**Conclusão** Pacientes com câncer de endométrio associado a pólipos podem ter o tumor completamente removido durante a histeroscopia, mas, com as variáveis avaliadas, é difícil predizer com segurança qual paciente ficará sem tumor residual.

## Introduction

Patients with endometrial cancer (EC) present with polyps at the time of diagnosis between 10 and 30% of the cases. 1 2 If the tumor originates from a typical polyp, it is described as a neoplastic polyp, but if the cancer acquires a polypoid configuration, it is classified as a polypoid cancer. 2 Patients with concomitant endometrial polyps and cancer usually present in earlier stages than those with tumors that are not associated with a polyp. 1

Molecularly, the epithelium of the polyp differs from that of the endometrium, 3 4 but can be as prone to carcinogenesis as the normal endometrium. 1 Although the risk factors for endometrial neoplasms are the same for polyps and normal endometrium, 1 5 the histological findings of the polyp might not reflect the condition of the adjacent endometrium. 5

Few studies have described the aggressiveness of a tumor that is associated with polyps. 1 6 In the case of EC in a polyp, the thickness of the parenchyma of the polyp can be a protective factor against myometrial invasion, based on reports of the absence of residual disease after polypectomy in patients with EC. 1 2

Atypical hyperplasia in a polyp usually requires a more conservative treatment. 7 8 Even histologically high-grade cancer that originates in a polyp can present as confined uterine disease 9 and thus receive less adjuvant treatment. 10 Moreover, endometrioid tumors appear to be less aggressive when they are restricted to a polyp, 11 but studies that have characterized such tumors are lacking.

Our aim was to correlate the location of endometrioid EC (within the polyp and/or the adjacent endometrium) with clinical and pathological factors, trying to find a subgroup of patients prone to the absence of residual neoplasia in the final uterine specimen.

## Methods

We analyzed a sequential series of 104 patients (92 cases from the Hospital AC Camargo and 12 from the Hospital do Servidor Público Estadual de São Paulo) who had presented endometrial polyps in the hysteroscopy and undergone surgery for EC from January 2002 to January 2017. Clinical and pathological data were retrospectively retrieved from their medical records. The respective institutional ethical review boards approved the present study (approval CAAE numbers 70580117.9.1001.5463/ 70580117.9.2001.5432).

We included patients with polyps and a diagnosis of EC. The diagnosis was achieved by hysteroscopic biopsy/polypectomy or defined after hysterectomy, based on the definitive pathologic uterine specimen. Patients with nonendometrioid tumors were excluded. Staging and grade were defined according to the International Federation of Gynecology and Obstetrics (FIGO) classification. 12

A database was constructed using SPSS for Mac, version 20.0 (SPSS, Inc., Chicago, IL, USA). The chi-squared and Fisher exact tests were used to analyze the correlations between categories and clinicopathological variables. For all tests, an α error of up to 5% ( *p* < 0.05) was considered significant.

## Results

The clinical and pathological characteristics are summarized in Table 1 . Menopause was described in 87 patients (83.7%), and the main symptom was vaginal bleeding. A high percentage of patients had lymph node dissection (72%), but no cases of lymph node involvement were observed.

**Table 1:** Clinical and pathological variables of the 104 patients with a diagnosis of endometrial polyps and cancer

Clinical variables	n = 104	%
Age - years old (mean)	58	
Menopause	87	83.7
Bleeding	61	58.1
Lymph node dissection	75	72.1
Absence of residual tumor	18	17.3

Table 2 lists the pathological variables according to the location of the tumor. In 78 cases (75%), the EC involved the polyp and the adjacent endometrium and in 40 (38.5%), it was restricted to the polyp, with no endometrial involvement. Moreover, in 16 cases (15.3%), the EC involved only the adjacent endometrium. In 10 (9.6%) cases, the polyp biopsy showed atypical hyperplasia and EC was detected only in the final pathological specimen. There was no correlation between the presence of postmenopausal bleeding and the exact location of the EC ( *p* = 0.42).

**Table 2:** Symptoms and hysteroscopic findings of the 104 patients with a diagnosis of endometrial polyps and cancer

Data	Status	Polyp involvement ( *n* = 40)	Polyp and endometrial involvement ( *n* = 38)	Endometrial involvement ( *n* = 16)	Neoplasm only in final specimen (10)	*p-value*
Bleeding	Yes No	24 16	25 13	8 8	4 6	0.42
Number of polyps	1 2 3+ Not described	17 6 4 13	16 2 6 14	8 0 0 8	3 3 0 4	0.25
Polyp procedure	Excision Biopsy not described	18 3 19	28 4 6	7 1 8	4 2 4	0.59

Most cases ( *n* = 96; 92.3%) were classified as stage IA and 90 (86.5%) were classified as grade 1 or 2. In 18 cases (17.3%), there was no residual tumor in the definitive hysterectomy specimen ( Table 3 ); however, of the 40 cases in which the tumor was apparently restricted to the polyp, 5 (12.5%) showed deep myometrial invasion in the definitive uterine specimen.

**Table 3:** Final pathological report variables of the 104 patients with a diagnosis of endometrial polyps and cancer

Data	Status	Polyp involvement ( *n* = 40)	Polyp and endometrial involvement ( *n* = 38)	Endometrial involvement ( *n* = 16)	Neoplasm only in final specimen ( *n* = 10)	Total	*p-value*
Residual tumor	Absent Present	9 31	6 32	3 13	0 10	18 86	0.45
Stage	IA IB	35 5	35 3	16 0	10 0	96 8	0.48
Grade	1 2 3	18 14 8	22 12 4	10 4 2	9 1 0	59 31 14	0.32

Cancer that was localized in the polyp by hysteroscopy was unrelated to a higher percentage of final specimens without disease ( Table 3 ). Even when cases with tumor involvement outside of the polyp were grouped, there was no significant impact on the presence of residual disease ( *p* = 0.20). Moreover, there was no significant association between polyp-restricted tumors and early-stage disease ( *p* = 0.13). Grade 3 tumors occurred irrespective of the precise location of the neoplasm. Of the 14 high-grade tumors (13.4%), 3 were IB tumors ( Table 3 ). Complete removal (with no residual disease) of the 14 grade 3 tumors by hysteroscopy was achieved in 5 patients (35.6%) and in 13 of the 90 grade grade 1 or 2 tumors (14.4%) ( *p* = 0.064).

## Discussion

Several studies have evaluated concomitant EC and endometrial polyps, 1 4 13 but this group of patients must be better characterized, aiming to tailor surgery and adjuvant treatment. The present study was performed at 2 cancer centers, where besides oncologic treatment, hysteroscopies are also usually performed to diagnose endometrial diseases.

The mean age and menopausal status of our patients were similar to those in previous studies. 14 15 16 We recorded a higher rate of asymptomatic patients, compared with previous studies, 17 18 concluding that hysteroscopic investigation of polyps can identify cases of initial EC, even in patients who still have no symptoms, such as vaginal bleeding. 1 19 20 Indeed, cancer that is associated with polyps appears to present at earlier stages, compared with those that do not, 1 but this probably does not cause an impact on survival rates. 21

The diagnosis of cancer as an unexpected finding occurred in 9.6% of the patients. They had undergone a pathological examination of polyps, which suggested a benign alteration (atypical hyperplasia), but the definitive pathological report described adenocarcinoma. This finding has been corroborated by other studies. 7 8 22 This situation was less frequent than that reported in the GOG 167, in which the prevalence of endometrial cancer after hysterectomy with atypical hyperplasia was 42.6%. 23 Notably, a systematic review confirmed that when the atypical hyperplasia is restricted to polyps, < 10% of the final specimen would be expected to have cancer. 7

At least 10 studies have evaluated definitive pathological reports of patients with an endometrial tumor that involved polyps ( Table 4 ). The criteria for patient selection varied significantly among studies, with some having a hysteroscopic focus 1 24 25 and others evaluating oncological outcomes and detailing the staging and follow-up. 2 5 6 26 27 28 29

**Table 4:** Previous studies with endometrioid uterine neoplasms associated with polyps

	n	n (%) Restricted to polyps in hysteroscopy	Grade 1 or 2	Stage IA (n/%)	Absence of residual tumor in hysterectomy
Salm (1972) 29 *	4	ND	4	ND	1 (25%)
Farrell et al. (2005) 2 *	26	ND	25 (96.1%)	26 (100%) (selected just IA)	6 (23%)
Fernández-Parra et al. (2006) 25	10	10	ND	8 (80%)	3 (30%)
Giordano et al. (2007) 26 *	5	5 (100%)	5 (100%)	3 (60%)	0 (0%)
Vilos et al. (2007) 28	10	10 (100%)	10	10 (100%)	2 (20%)
Mittal et al. (2008) 5 *	18	11 (61%)	ND	16 (88.9%)	1 (5.6%)
Perri et al. (2010) 1 **	125	ND	96 (76.8%)	96 (76.8%)	13 (10.4%)
Wethington et al. (2011) 24 **	13	3 (23.1%)	ND	ND	ND
Gambadauro et al. (2014) 6 *	12	ND	11 (91.6%)	8 (66.7%)	0 (0%)
Elyashiv et al. (2017) 27	18	ND	ND	13 (72.2%)	4 (22.2%)
Present study	104	40 (38.5%)	86.5	96 (92.3%)	18 (17.3%)

Abbreviation: ND, not described.

* Other histological types were excluded from this table.

** Other histological types included in the analysis.

In our series, when the polyp was associated with cancer, the tumor in most cases involved the polyp, (78 cases, 75%), but in 38.5%, it was restricted to the polyp and in 36.5%, there was also endometrial involvement. With our findings, it was not possible to establish whether the malignancy is more likely to affect the polyp tissue or the adjacent endometrium. Conversely, our data suggest that the tumor randomly affects the adjacent polyp or the endometrium. Similarly, Farrell et al. 2 reported the involvement of the adjacent endometrium in 61.5% (16/26) of cases and 6 cases had no residual disease in the final pathologic uterine specimen (23%).

Complete removal of the polyp when the tumor was restricted to the polyp was achieved in 18/40 cases (45%), but only in 9 (50%) of them it resulted in a uterine specimen without cancer. This finding suggests that hysteroscopy is not very accurate for distinguishing neoplastic tissue and that the complete resection of the polyp does not always match the definitive pathological result. 27

It is possible that an early neoplasm, opportunistically diagnosed in asymptomatic patients, allows for the tumor to be resected entirely by hysteroscopy. If the disease develops, it is expected that tumors that are initiated in pre-existing polyps will affect the base of the polyp and the endometrium. In this context, it would no longer be possible to distinguish the neoplastic polyp from a polypoid neoplasm. 2

It is more difficult for extra-polyp disease to be removed by hysteroscopy, but 6 (15.7%) of 38 patients in this group had a definitive uterine specimen without cancer. Removal of all EC tissue by hysteroscopy was reported previously in some exceptional situations. 28 30

Almost all the series described tumors with stages > IA ( Table 4 ), with the prevalence ranging between 0 and 30%, and some of them have also described stages II-IIIA. 1 6 26 Although in the present study and in most of the previous series there was no lymph node involvement, one previous study documented stages III and IV, 1 suggesting that lymph node evaluation could not be omitted. We excluded nonendometrioid types from our analysis, due to the particularly aggressive behavior of the disease in this group. 2

The inherent nature of a retrospective study hindered a more precise hysteroscopic characterization of the biopsy site, especially the location of the tumor in the polyp. Conversely, several years would be necessary to recruit this sample in a prospective study.

Because blind biopsies usually have a lower diagnostic accuracy, 31 32 a potential bias of our study is that, in certain cases, the biopsy was performed after a diagnostic hysteroscopy with curettage, rather than with a working instrument. Nevertheless, blind avulsion of the polyp using the appropriate materials and standardized technique leads to an adequate pathological analysis. 33 In the case of curettage, the sample fragmentation makes it impossible to differentiate whether the biopsy came from the polyp or the endometrium. 34

Another concern is that certain patients with polypoid cancer were inadvertently included, due to poor hysteroscopic descriptions when the exam was performed outside of the referred services. In some hysteroscopies, only the appearance of the polyp, and not that of the adjacent endometrium, was described.

The absence of pathological indicators of the low aggressiveness of tumors that are restricted to polyps is our most important finding. The strength of our study is its large sample size in comparison with other studies on polyps and cancer, and our data confirm that even patients with endometrioid EC that is apparently restricted to the polyp in hysteroscopy, regardless of histological grade, can experience deep myometrial invasion.

## Conclusion

In conclusion, there was no association between polyp location of the EC during hysteroscopy and less residual disease or absence of deep myometrial invasion in the final uterine specimen.
